# Transplantation of Melanocytes Obtained from the Skin Ameliorates Apomorphine-Induced Abnormal Behavior in Rodent Hemi-Parkinsonian Models

**DOI:** 10.1371/journal.pone.0065983

**Published:** 2013-06-12

**Authors:** Masato Asanuma, Ikuko Miyazaki, Francisco J. Diaz-Corrales, Youichirou Higashi, Masayoshi Namba, Norio Ogawa

**Affiliations:** 1 Department of Brain Science, Okayama University Graduate School of Medicine, Dentistry and Pharmaceutical Sciences, Okayama, Japan; 2 Niimi College, Niimi, Japan; University G. D'Annunzio, Italy

## Abstract

Tyrosinase, which catalyzes both the hydroxylation of tyrosine and consequent oxidation of L-DOPA to form melanin in melanocytes, is also expressed in the brain, and oxidizes L-DOPA and dopamine. Replacement of dopamine synthesis by tyrosinase was reported in tyrosine hydroxylase null mice. To examine the potential benefits of autograft cell transplantation for patients with Parkinson’s disease, tyrosinase-producing cells including melanocytes, were transplanted into the striatum of hemi-parkinsonian model rats or mice lesioned with 6-hydroxydopamine. Marked improvement in apomorphine-induced rotation was noted at day 40 after intrastriatal melanoma cell transplantation. Transplantation of tyrosinase cDNA-transfected hepatoma cells, which constitutively produce L-DOPA, resulted in marked amelioration of the asymmetric apomorphine-induced rotation in hemi-parkinsonian mice and the effect was present up to 2 months. Moreover, parkinsonian mice transplanted with melanocytes from the back skin of black newborn mice, but not from albino mice, showed marked improvement in the apomorphine-induced rotation behavior up to 3 months after the transplantation. Dopamine-positive signals were seen around the surviving transplants in these experiments. Taken together with previous studies showing dopamine synthesis and metabolism by tyrosinase, these results highlight therapeutic potential of intrastriatal autograft cell transplantation of melanocytes in patients with Parkinson’s disease.

## Introduction

Similar to the peripheral melanin biosynthetic pathway, the multifunctional enzyme tyrosinase (EC 1.14.18.1; monophenol monooxygenase) in the brain catalyzes the conversion of L-tyrosine to L-3,4-dihydroxyphenylalanine (L-DOPA) and oxidizes L-DOPA to form dopa quinone, which is a melanin precursor. In addition, it is known that tyrosinase also oxidizes dopamine (DA) to rapidly form melanin via the DA quinone [1]. Several studies have demonstrated the expression, promoter activity of tyrosinase, and tyrosinase-like enzyme activity in the substantia nigra [2], [3], [4]. Furthermore, a previous study has demonstrated that DA quinone, the formation of which is enhanced by tyrosinase, inactivates tyrosine hydroxylase (TH) [5]. On the other hand, supplement of DA synthesis by tyrosinase has been reported in TH null mice [6]. These findings imply that tyrosinase may be involved in catecholaminergic cell function, and that the activity of this enzyme may contribute to the pathogenesis or complementary reaction associated with Parkinson's disease (PD).

The aim of present study was to investigate the effects of transplantation of tyrosinase-producing cells in parkinsonian models. Different approaches were used for this purpose. *1)* B16-F1 melanoma cells or non-melanogenic cells transfected with tyrosinase gene, as tyrosinase-producing cells, were transplanted into the striatum of hemi-parkinsonian models lesioned with 6-hydroxydopamine (6-OHDA). Following these approaches, we then examined changes in apomorphine-induced contralateral rotation behavior and in striatal expression of DA and tyrosinase. *2)* Finally, we examined transplantation of melanocytes from the back skin, as clinically available tyrosinase-expressing cells, into the striatum of hemi-parkinsonian mice.

## Materials and Methods

### Materials and Animals

The chemicals listed below were purchased from Sigma-Aldrich (St. Louis, MO): 6-OHDA hydrobromide, apomorphine, dibutyryl cAMP, insulin, bovine serum albumin, ethanolamine, phosphoethanolamine, sodium selenite, gentamicin sulfate, amphotericin B and 3,3′-diaminobenzidine (DAB). Male Sprague-Dawley rats weighing 280–300 g (9-weeks-old) and male ICR mice weighing 30–35 g (8-weeks-old) were purchased from Charles River Japan, Yokohama, Japan and used to establish an animal model of hemi-parkinsonism. Newborn albino ICR mice or black C57BL mice were used for primary cultures of melanocytes. All animal procedures were in strict accordance with the Guideline for Animal Experiments of Okayama University Advanced Science Research Center, and were approved by the Animal Care and Use Committee of Okayama University Advanced Science Research Center. Special interest was taken to minimize the number of animals used in this research.

### Hemi-parkinsonian Rats

Unilateral nigrostriatal dopaminergic lesions were generated using the method described previously with some modifications [7]. Briefly, 6-OHDA hydrobromide (5 µg in 2 µl saline containing 0.1% ascorbate) was injected at two sites (5 µg/site) in the right medial forebrain bundle of male Sprague-Dawley rats (9-weeks-aged) under pentobarbital anesthesia (50 mg/kg, i.p.) at the following coordinates: A −1.0 mm, L +1.8 mm, V +8.5 mm; A −1.4 mm, L +1.5 mm, V +8.0 mm from the bregma according to the atlas of the rat brain (upper incisor bar set 5.0 mm above the interaural line [8]). Sham-operated control rats were injected with the same volume of saline containing 0.1% ascorbate.

### Intrastriatal Injection of Liposome-entrapped Tyrosinase

At 14 and 34 days after injection of 6-OHDA into the right medial forebrain bundle, apomorphine (0.1 mg/kg, s.c.) rotation test was performed to confirm the lesion of nigrostriatal dopaminergic pathway. Rats that exhibited asymmetric rotation behavior towards the contralateral side of >10 turns/2 min at 10 min after injection of apomorphine were selected for the subsequent tyrosinase replacement study. Liposome-entrapped mushroom tyrosinase [9], [10] (19.2 U/4 µg/4 µl lipofectin) or lipofectin alone was injected at two sites (4 µl/site) in the right lesioned side of the lateral striatum of hemi-parkinsonian rats as well as sham-operated control rats under pentobarbital anesthesia (50 mg/kg, i.p.) at the following coordinates: A +0.7 mm, L +3.0 mm, V +4.0 mm and +5.0 mm from the bregma according to the atlas of the rat brain (upper incisor bar set 3.9 mm below the interaural line; [11]) at 38 days after 6-OHDA lesioning. Apomorphine (0.1 mg/kg, s.c.) rotation test was performed at 3, 6 and 12 days after tyrosinase injection. Rotation behavior towards the contralateral side over a 2-min period was observed at 10 min after apomorphine injection.

### Intrastriatal Transplantation of B16-F1 Melanoma Cells

Lesioning by 6-OHDA was confirmed by apomorphine (0.1 mg/kg, s.c.) rotation test at 16 and 43 days after injection into the right medial forebrain bundle. Rats that exhibited asymmetric rotation behavior towards the contralateral side of >20 turns/5 min at 5 min after apomorphine injection were selected for the subsequent melanoma cell transplantation. The mouse B16-F1 melanoma cells (ATCC # CRL-6323, Manassas, VA) were cultured in Dulbecco’s Modified Eagle Medium (DMEM; Gibco BRL, Rockville, MD) supplemented with 10% (v/v) fetal bovine serum (FBS), 4 mM L-glutamine, 100 U/ml penicillin and 100 µg/ml streptomycin. Under pentobarbital anesthesia (50 mg/kg, i.p.), B16-F1 melanoma cells suspended with PBS (5×10^5^ cells/4 µl PBS) or the same volume of PBS as control were transplanted at two sites (4 µl/site) in the right lateral striatum of hemi-parkinsonian rats as well as saline-injected sham-operated rats at 46 days after 6-OHDA lesioning at the following coordinates: A +0.7 mm, L +3.0 mm, V +4.0 mm and +5.0 mm from the bregma according to the atlas of the rat brain (upper incisor bar set 3.9 mm below the interaural line; [11]). Apomorphine (0.1 mg/kg, s.c.) rotation test was performed at 3, 7, 14 and 40 days after B16-F1 melanoma cell transplantation. Rotation behavior towards contralateral side over a period of 5 min was examined at 5 min after apomorphine injection.

### Hemi-parkinsonian Mice

To prepare hemi-parkinsonian mice, unilateral striatal lesions were induced by intrastriatal injection of 6-OHDA with some modification of the previously reported procedure [12]. Ten µg of 6-OHDA in 1 µl of physiological saline containing 0.1% ascorbic acid was injected into two sites (10 µg 6-OHDA/site) in the right striatum of male ICR mice under pentobarbital anesthesia (50 mg/kg, i.p.) at the following coordinates: A +0.7 mm, L +2.0 mm, V +3.0 mm; A +0.2 mm, L +2.4 mm, V +3.0 mm from the bregma according to the atlas of the mouse brain (upper incisor bar set 2.0 mm below the interaural line) [13]. Sham-operated control mice were injected with the same volume of saline containing 0.1% ascorbate.

### Intrastriatal Transplantation of Tyrosinase Gene-transfected Hepatoma Cells

After the above mentioned 6-OHDA lesioning in mice, apomorphine (0.5 mg/kg, s.c.) rotation test was performed at Days 17 and 24 to confirm the lesion. Mice that exhibited asymmetric rotation behavior towards the contralateral side of >30 turns/10 min after apomorphine injection were selected for subsequent transplantation. HLE cell line, a commonly used human hepatoma cell line which we first established [14], [15], [16], was cultured in DMEM supplemented with 10% FBS and 100 µg/ml kanamycin, and then transfected with human tyrosinase cDNA (HLE/tyrosinase) or empty vector (HLE) as reported previously [17]. The transfected cells were suspended with PBS (1×10^6^ cells/2 µl), and then transplanted into the right lateral striatum of hemi-parkinsonian albino mice under pentobarbital anesthesia (50 mg/kg, i.p.) at 28 days after the 6-OHDA lesioning at the following coordinate: A +0.5 mm, L +2.0 mm, V +3.0 mm from the bregma (upper incisor bar set 2.0 mm below the interaural line) [13]. Apomorphine (0.5 mg/kg, s.c.) rotation test was performed at 3–77 days after HLE/tyrosinase transplantation. Rotation behavior towards the contralateral side over a period of 10 min was examined after apomorphine injection.

### Intrastriatal Transplantation of Melanocytes

In apomorphine (0.5 mg/kg, s.c.) rotation test at 28 and 44 days after 6-OHDA injection conducted to confirm 6-OHDA-induced lesion, albino ICR mice that exhibited asymmetric rotation behavior towards the contralateral side of >30 turns/10 min after injection were selected for the subsequent transplantation of melanocytes. Melanocytes were harvested from the back of newborn albino ICR mice or black C57BL mice by filtration through 40 µm-nylon mesh according to the previously reported method [18], [19], and then cultured in melanocyte culture medium (MDMD: melanoblast-defined medium with dibutyryl cAMP) consisting of Ham’s F10 medium (Gibco BRL) supplemented with 10 µg/ml insulin, 1 mg/ml bovine serum albumin, 1 µM ethanolamine, 1 µM phosphoethanolamine, 10 nM sodium selenite, 50 µg/ml gentamicin sulfate, 0.25 µg/ml amphotericin B and 0.5 mM dibutyryl cAMP. Melanocytes from albino or black mice suspended with medium (3×10^5^ cells/2 µl) were transplanted into the right lateral striatum of hemi-parkinsonian albino mice as well as saline-injected sham-operated controls under pentobarbital anesthesia (50 mg/kg, i.p.) on Day 48 after 6-OHDA lesioning at the following coordinate: A +0.5 mm, L +2.0 mm, V +3.0 mm from the bregma (upper incisor bar set 2.0 mm below the interaural line) [13]. Apomorphine (0.5 mg/kg, s.c.) rotation test was performed at 3–86 days after melanocyte transplantation. The rotation behavior towards contralateral side over a period of 10 min was conducted after apomorphine injection.

### Immunohistochemistry of Hemi-parkinsonian Brain

Animals were transcardially perfused with 4% paraformaldehyde at 3 or 14 days after tyrosinase lipofection, at 52 days after intrastriatal B16-F1 melanoma cell transplantation, at 80 days after HLE/tyrosinase transplantation, and at 88 days (12 weeks) after intrastriatal melanocyte transplantation. The perfused brains were removed and post-fixed for 24 h in 4% paraformaldehyde. Following cryoprotection in 15% sucrose in PB for 48 h, the brains were snap-frozen with powdered dry ice and 20-µm-thick coronal sections were cut on a cryostat at the mid-striatal level through the tyrosinase-injected area or around the transplant and at level of the substantia nigra pars compacta.

Striatal tyrosinase- or DA-immunoreactivity in transplanted animals was visualized by fluorescent immunohistochemistry using goat polyclonal anti-tyrosinase antibody (dilution 1∶200, Santa Cruz Biotechnology, Santa Cruz, CA) or rabbit polyclonal anti-DA antibody (dilution 1∶200, Chemicon International, Temecula, CA) and FITC-conjugated anti-goat IgG or rhodamine-conjugated anti-rabbit IgG secondary antibody (Chemicon), respectively. Double immunofluorescence staining of tyrosinase and TH or glial fibrillary acidic protein (GFAP) in mid-striatal or nigral sections of hemi-parkinsonian rats were also performed using anti-tyrosinase antibody (dilution 1∶200, Santa Cruz Biotechnology) and rabbit anti-TH polyclonal antibody (dilution 1∶10,000, Protos Biotech, New York, NY) or rabbit anti-GFAP antibody (1∶1,000; Dako Cytomation, Glostrup, Denmark). Sections were also stained by hematoxylin-eosin to visualize the transplant. Tyrosinase-positive (green) and DA-positive, TH-positive or GFAP-positive (red) immunofluorescence signals were analyzed under a fluorescence microscope (Olympus BX50-FLA, Tokyo) using a mercury lamp through a 470–490 nm or 530–550 nm, band-pass filter to excite FITC or rhodamine, respectively. Light emitted from FITC or rhodamine was collected through 515–550 nm band-pass filter or 590 nm long-pass filter, respectively.

TH-immunoreactivity (IR) was also visualized in striatal or nigral sections of transplanted animals by standard free-floating immunohistochemistry with chromogen DAB. The sections were then incubated with anti-TH rabbit polyclonal antibody (dilution 1∶10,000, Protos Biotech, New York, NY) overnight at 4°C, followed by incubation for 2 h at room temperature with biotinylated goat anti-rabbit IgG secondary antibody (dilution 1∶1,000; Vector Laboratories, Burlingame, CA). After washing, the sections were incubated with avidin-biotin peroxidase complex (dilution 1∶2,000, Vector) for 1 h at room temperature. TH-IR was visualized by DAB, nickel, and hydrogen peroxide. Nuclear counter-staining was performed with methylgreen.

The relative density of tyrosinase-, DA- or TH-IR was semi-quantified using a microscope in at x200 magnification for the mid-striatum and x100 magnification for the substantia nigra pars compacta and a Macintosh computer-based image analysis system (NIH Image J 1.44k) [20]. For precise measurement, the mid-striatum was divided into two areas of lateral striatum and medial striatum. The relative density of immunopositive signals in the lateral striatum was quantitatively measured. The boundary between the substantia nigra pars compacta and ventral tegmental area was defined by a line extending dorsally from the most medial boundary of the cerebral peduncle.

### Fluorescent Immunocytochemistry of Tyrosinase Gene-transfected Cells

Cultured HLE cells transfected with tyrosinase cDNA (HLE/tyrosinase) on 4-chamber culture slides (Becton Dickinson, Franklin lakes, NJ) were fixed with 4% paraformaldehyde. Tyrosinase- or L-DOPA-IR was detected by immunohistochemistry using goat polyclonal anti-tyrosinase antibody (dilution 1∶200, Santa Cruz Biotechnology) or rabbit polyclonal anti-L-DOPA antibody (dilution 1∶200, Chemicon) and FITC-conjugated anti-goat IgG or rhodamine-conjugated anti-rabbit IgG secondary antibody (Chemicon), respectively. The nuclei were counterstained with 10 µg/ml Hoechst 33342 (Invitrogen, San Diego, CA). Immunofluorescence signals were analyzed under a fluorescence microscope as mentioned above.

### Measurement of DA and its Metabolites in Striatum of Hemi-parkinsonian Mice

Striatal tissue of hemi-parkinsonian mice was removed at 80 days after transplantation of HLE cells transfected with tyrosinase cDNA (HLE/tyrosinase). The contents of L-DOPA, DA and its metabolites, 3,4-dihydroxyphenyl acetic acid (DOPAC) and homovanillic acid (HVA), were measured using high-performance liquid chromatography with an electrochemical detector (HPLC-ECD) as described previously [7]. The striatal tissue was homogenized with 5 volumes of 200 mM ice-cold perchloric acid containing 10 mM EDTA. After centrifugation (11,750×g, for 20 min at 4°C), the supernatant was filtered (0.45 µm) and then injected directly into a HPLC-ECD (Tosoh Co., Tokyo, Japan). The HPLC system consisted of a delivery pump (PX-8020, Tosoh Co.) and an analytical column (EICOMPAK SC-5ODS, 3.0 mm×150 mm, Eicom Co., Kyoto, Japan). An electrochemical detector (EC-8020, Tosoh Co.) with glassy carbon was used with a voltage setting of 700 mV and an Ag/AgCl reference electrode. A mobile phase containing 0.1 M citrate-sodium acetate buffer (pH 3.5), methanol (17% v/v), EDTA-2Na, and sodium 1-octanesulfonate was infused at a flow rate of 0.6 ml/min.

### Statistical Analysis

Values were expressed as means ± SEM. Differences between groups were examined for statistical significance by one-way or two-way analysis of variance (ANOVA), followed by post hoc Fisher’s PLSD multiple comparison test. A *P* value less than 0.05 was considered statistically significant.

## Results

### Expression of Tyrosinase in the Basal Ganglia of Hemi-parkinsonian Rats

**Figure 1:**
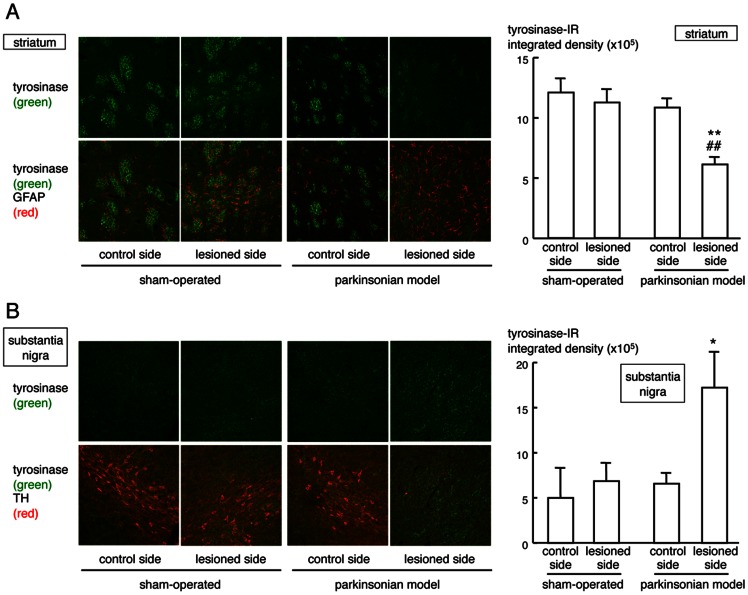
Expression of tyrosinase in the basal ganglia of hemi-parkinsonian rats. (A) Immunofluorescence signals using anti-tyrosinase antibody (green) and anti-GFAP antibody (red) in the mid-striatum on the control side or lesioned side of hemi-parkinsonian rats and sham-operated controls at 4 weeks after lesioning by 6-OHDA injection. (B) Double immunofluorescence staining using anti-tyrosinase antibody (green) and anti-TH antibody (red) in the substantia nigra of the intact side or lesioned side of hemi-parkinsonian rats and sham-operated controls. (A, B) Each right panel shows means integrated density of tyrosinase-IR ± SEM (n = 3). *p<0.05, **p<0.01 vs. intact side of each treated group. ^##^ *p*<0.01 vs. side-matched sham-operated control group.

We examined changes in tyrosinase expression in the basal ganglia of hemi-parkinsonian rats. Tyrosinase-positive signals were increased on the lesioned side of the substantia nigra, but decreased on the lesioned side of the striatum at 4 weeks after 6-OHDA injection (Figure 1 and Figure S1B). The increase in nigral tyrosinase expression implies complementary DA synthesis by tyrosinase in the parkinsonian models we used as well as TH null mice [6].

### Intrastriatal Injection of Liposome-entrapped Tyrosinase in Hemi-parkinsonian Rats

Asymmetric rotation behavior towards the contralateral side was seen in 6-OHDA-injected hemi-parkinsonian model rats after apomorphine injection. The apomorphine-induced rotation behavior of hemi-parkinsonian model was significantly but transiently improved at 3 and 6 days after replacement of liposome-entrapped mushroom tyrosinase (19.2 U/4 µg/4 µl lipofectin X2) into the lesioned side of the striatum (Figure S1A and Movie S1). Increased expression of tyrosinase-positive signals on the injected (lesioned) side of the striatum was lasted at 14 days after injection of liposome-entrapped tyrosinase in both sham-operated and hemi-parkinsonian animals compared with the injected side in lipofectin-injected mice or the non-injected (control) side in each tyrosinase-injected mice (Figure S1B). Marked increase in DA-IR was also noted on the injected side of the striatum at 14 days after tyrosinase injection, especially in hemi-parkinsonian rats (Figure S1C). However, the intrastriatal injection of liposome-entrapped tyrosinase showed no effects on TH-IR in the basal ganglia (Figure S2).

### Intrastriatal Transplantation of B16-F1 Melanoma Cells in Hemi-parkinsonian Rats

**Figure 2:**
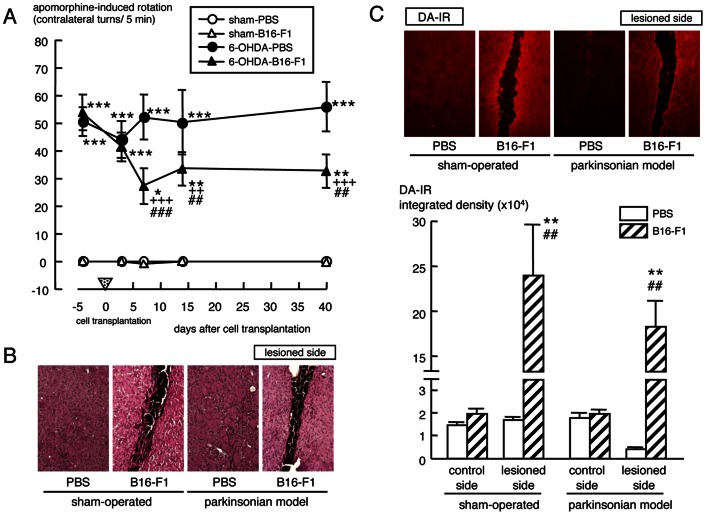
Effects of intrastriatal transplantation of B16-F1 melanoma cells in hemi-parkinsonian rats. (A) Rotation behavior towards contralateral side for 5 min at 5 min after injection of apomorphine (0.1 mg/kg, s.c.) was tested at 3, 7, 14 and 40 days after transplantation of B16-F1 melanoma cell (5×10^5^ cells/4 µl PBS X2) into the right lateral striatum of hemi-parkinsonian rats or sham-operated controls. Values are means ± SEM of 3–4 rats. *p<0.05, **p<0.01, ***p<0.001 vs. each sham-PBS, ^++^ *p*<0.01, ^+++^ *p*<0.001 vs. each 6-OHDA-PBS, ^##^ *p*<0.01, ^###^ *p*<0.001 vs. each pre-transplantation of B16-F1 cells. Representative photographs of hematoxylin-eosin-staining (B) and DA-IR (C) of the striatal area around the transplant at 52 days after intrastriatal transplantation of B16-F1 melanoma cells in hemi-parkinsonian rats and sham-operated controls. (C) Lower panel shows means integrated density of DA-IR ± SEM (n = 3–4). **p<0.0001 vs. control side of each treated group. ^##^ *p*<0.0001 vs. side-matched PBS-injected control group.

The apomorphine-induced rotation behavior towards the contralateral side was significantly and markedly improved in hemi-parkinsonian rats at days 7 and 14 after intrastriatal transplantation of B16-F1 melanoma cells (5×10^5^ cells/4 µl PBS X2), and such effect persisted up to 40 days after cell transplantation (Figure 2A and Movie S2). Dark melanoma cells were observed in the needle tract on the injected side of the striatum in both sham-operated and hemi-parkinsonian animals after cell transplantation (Figure 2B and Figure S3). The intrastriatal transplantation of B16-F1 melanoma cells significantly and markedly increased DA-IR around the surviving transplant on the injected side of the striatum at 52 days after transplantation in both sham-operated and hemi-parkinsonian animals (Figure 2C), without affecting TH-IR in the striatum and substantia nigra (Figure S3).

### Intrastriatal Transplantation of Tyrosinase Gene-transfected Hepatoma Cells in Hemi-parkinsonian Mice

**Figure 3:**
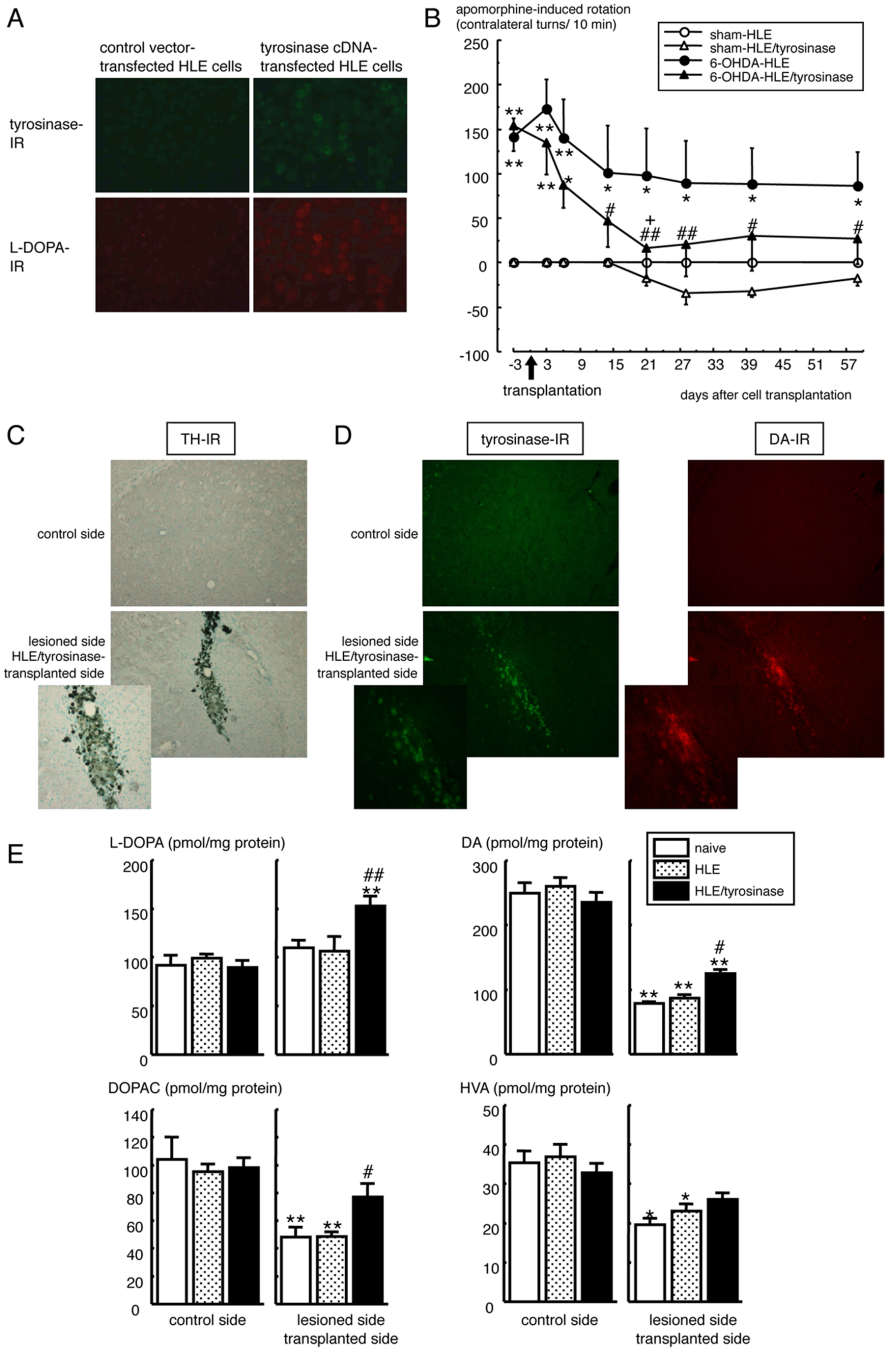
Effects of intrastriatal transplantation of tyrosinase cDNA-transfected hepatoma HLE in hemi-parkinsonian mice. Human hepatoma HLE cells transfected with tyrosinase cDNA (HLE/tyrosinase) (1×10^6^ cells/2 µl PBS) or empty vector (HLE) were transplanted into the right (lesioned side) lateral striatum of hemi-parkinsonian albino mice. (A) Double immunostaining of tyrosinase/L-DOPA in cultured human tyrosinase cDNA-transfected HLE cells. (B) Rotation behavior towards contralateral side over 10 min after injection of apomorphine (0.5 mg/kg, s.c.) was recorded at 3–77 days after HLE/tyrosinase transplantation. Values are means ± SEM of 4 mice. *p<0.05, **p<0.001 vs. each time-matched sham-HLE group. ^+^ *p*<0.05 vs. each time-matched 6-OHDA-HLE group. ^#^ *p*<0.01, ^##^ *p*<0.001 vs. each pre-transplantation of HLE cells. (C–D) Histological changes in the striatal area around the transplant after transplantation of intrastriatal HLE/tyrosinase into hemi-parkinsonian mice. TH-immunostaining with nuclear counter-staining using methylgreen (C), tyrosinase-positive IR (D; green) and DA-positive IR (D; red) in the right lateral striatum around the transplant of hemi-parkinsonian mice at 80 days (12 weeks) after intrastriatal HLE/tyrosinase transplantation. (E) Changes in the striatal contents of L-DOPA, DA and its metabolites DOPAC and HVA of hemi-parkinsonian mice at 80 days after HLE/tyrosinase transplantation. Values are means ± SEM of 6–8 samples from 3–4 mice. *p<0.001, **p<0.0005 compared with the control side of each treated group. ^#^ *p*<0.05, ^##^ *p*<0.005 compared with side-matched HLE cell-transplanted group.

Furthermore, non-melanogenic human hepatoma HLE cells transfected with tyrosinase cDNA (HLE/tyrosinase) were transplanted into the striatum of hemi-parkinsonian mice. Intrastriatal transplantation of empty vector-transfected non-melanogenic HLE cells that produce no tyrosinase or L-DOPA did not show any effects on apomorphine-induced rotation behavior towards the control side in hemi-parkinsonian model mice (Figure 3A, 3B and Movie S3). In the group transplanted with tyrosinase cDNA-transfected HLE cells that constitutively produced L-DOPA (Figure 3A), however, the apomorphine-induced rotation behavior towards the contralateral side was markedly improved at 14 days and up to 77 days (2 months) after transplantation (Figure 3B and Movie S3). High tyrosinase- and DA-IR was detected in surviving tyrosinase-expressing transplant on the injected side of the striatum of hemi-parkinsonian mice at 80 days after transplantation (Figure 3D). In the lesioned-side of the striatum where TH-IR was reduced, dark melanin granules were observed in the surviving transplanted tyrosinase cDNA-transfected HLE cells (Figure 3C). The level of L-DOPA was significantly increased in the injected side of the striatum at 80 days after intrastriatal HLE/tyrosinase transplantation, compared with naive or empty vector-transfected HLE cell-transplanted hemi-parkinsonian mice (Figure 3E). Furthermore, HLE/tyrosinase transplantation rectified the reduced levels of DA and DOPAC on the lesioned side of the striatum in hemi-parkinsonian animals.

### Intrastriatal Transplantation of Primary Cultured Melanocytes in Hemi-parkinsonian Mice

**Figure 4:**
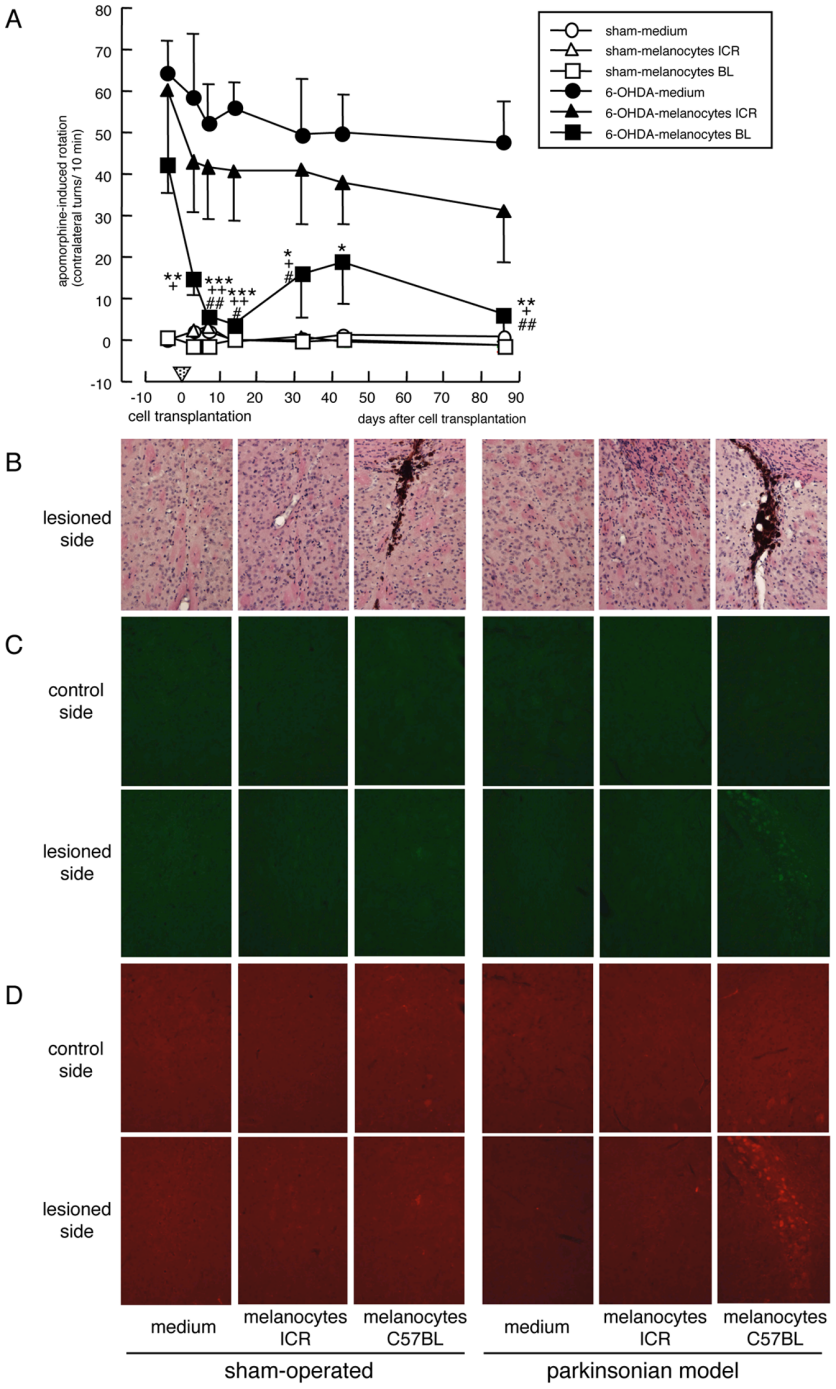
Effects of intrastriatal transplantation of primary cultured melanocytes in hemi-parkinsonian mice. Melanocytes from albino (ICR) or black (BL) mice suspended with medium (3×10^5^ cells/2 µl) were transplanted into the right lateral striatum of hemi-parkinsonian albino mice and sham-operated controls at 48 days after 6-OHDA lesioning. (A) Asymmetric rotation behavior towards contralateral side over 10 min after injection of apomorphine (0.5 mg/kg, s.c.) was recorded at 3–86 days after melanocyte transplantation. Values are means ± SEM of 3–4 mice. *p<0.05, **p<0.01, ***p<0.001 vs. each 6-OHDA-medium, ^+^p<0.05, ^++^p<0.01 vs. each 6-OHDA-melanocytes ICR, ^#^p<0.05, ^##^p<0.01 vs. each pre-transplantation of melanocytes. Histological changes in the striatal area around the transplant after intrastriatal melanocyte transplantation into hemi-parkinsonian mice. Hematoxylin-eosin-staining (B), tyrosinase-positive IR (C), and DA-positive IR (D) in the right lateral striatum around the transplant of hemi-parkinsonian albino mice and sham-operated controls at 88 days (12 weeks) after intrastriatal transplantation of melanocytes obtained from albino (ICR) or black (C57BL) mice.

**Figure 5:**
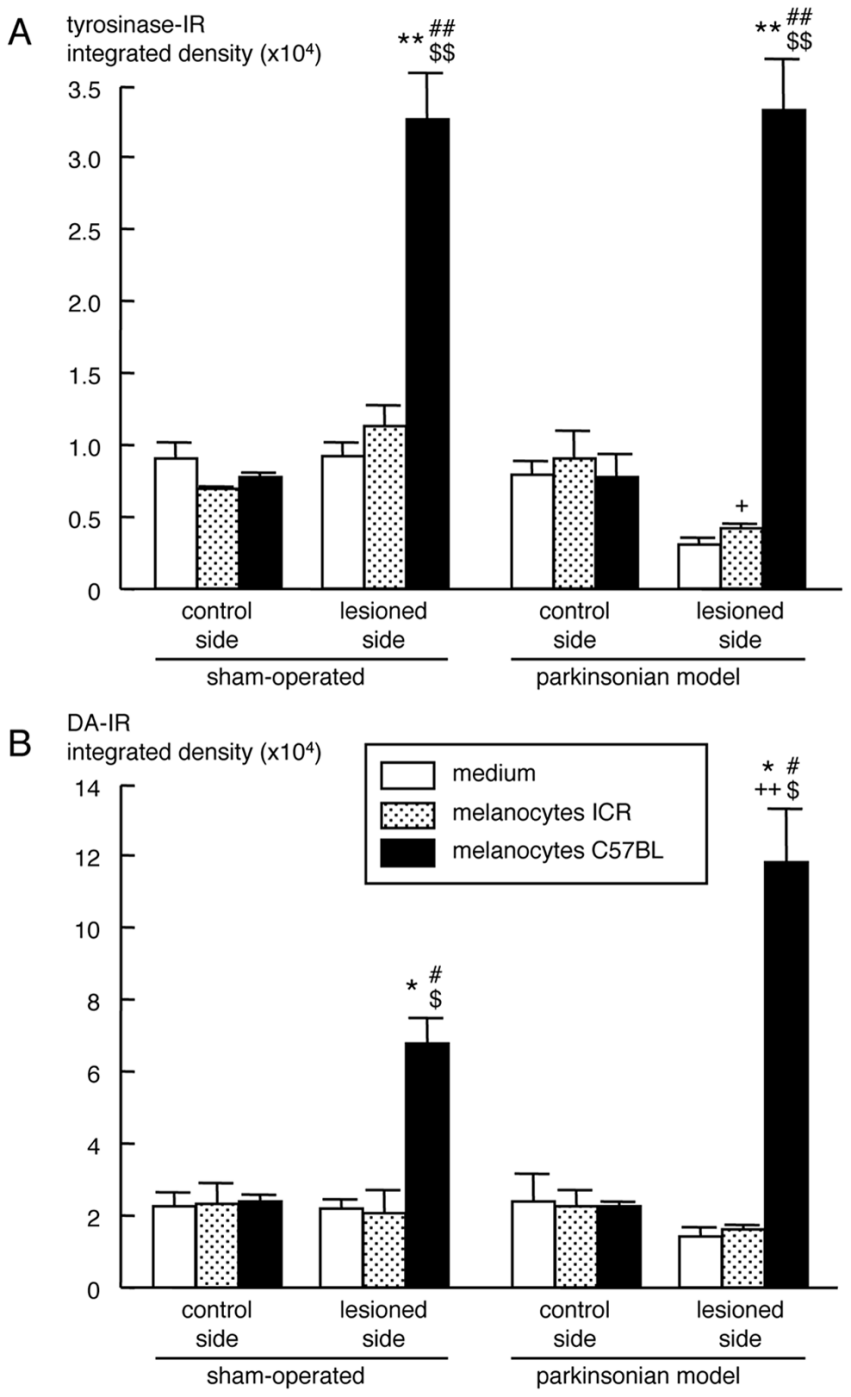
Tyrosinase-positive IR (A) and DA-IR (B) in the right lateral striatum around the transplant of hemi-parkinsonian albino mice and sham-operated controls at 88 days after intrastriatal transplantation of melanocytes obtained from albino (ICR) or black (C57BL) mice. Values are means integrated density of tyrosinase-IR or DA-IR ± SEM (n = 3–4). *p<0.005, **p<0.0001 vs. intact control side of each treated group. ^#^ *p*<0.005, ^##^ *p*<0.0001 vs. side-matched medium-injected control group. ^$^ *p*<0.005, ^$$^ *p*<0.0001 vs. side-matched melanocytes from ICR mice-transplanted group. ^+^ *p*<0.05, ^++^ *p*<0.0005 vs. side- and treatment-matched sham-operated.

In hemi-parkinsonian mice transplanted with primary cultured melanocytes obtained from the back skin of newborn C57BL black mice (tyrosinase activity: approx. 49 U/mg protein), the apomorphine-induced rotation behavior towards the contralateral side was markedly improved at 7–14 days up to 12 weeks (3 months) after transplantation, compared with transplanted group with melanocytes from albino ICR mice (tyrosinase activity: null) or control group injected with the medium only (Figure 4A and Movie S4). Even transplantation with melanocytes obtained from albino ICR mice had beneficial effects, though not significantly, on asymmetric rotation behavior. Surviving melanocytes containing dark melanin granules were observed on the injected side of the striatum in hemi-parkinsonian mice transplanted with melanocytes from black mice, but not from albino tyrosinase-null ICR mice (Figure 4B). Dramatic increases in expression of tyrosinase-IR (Figures 4C and 5A) and DA-positive signals (Figures 4D and 5B) were detected around the surviving transplant on the lesioned side of the striatum at 88 days (3 months) after the transplantation of melanocytes from black mice in hemi-parkinsonian animals. However, the transplantation of melanocytes showed no effects on TH-IR both in the striatum and substantia nigra (Figure S4).

## Discussion

The main findings of this study are: (1) transplantation of tyrosinase-producing cells into the striatum resulted in marked amelioration of the apomorphine-induced abnormal rotation behavior with increase in L-DOPA and DA in hemi-parkinsonian animal models, and (2) intrastriatal transplantation of melanocytes from the back skin as clinically available tyrosinase-expressing cells showed lasting improvement in the apomorphine-induced rotation behavior in hemi-PD model mice, with increase in DA-positive signals around the surviving transplants.

The melanin-synthesizing enzyme tyrosinase is expressed in the brain [2], [3], [4]. In impaired dopaminergic neuronal system, complementary DA synthesis by tyrosinase was reported in TH null mice [6]. In the present study, tyrosinase-positive signals were increased on the lesioned side of the substantia nigra pars compacta in hemi-parkinsonian animals (Figure 1). Therefore, the increase in tyrosinase in the substantia nigra may be a reaction to complement TH in the parkinsonian brain. Amicarelli et al. reported previously that intrastriatal injection of liposome-entrapped tyrosinase significantly increased the DA level in the striatum of normal rats [9]. Since the DA-positive signals was increased up to 14 days after intrastriatal injection of liposome-entrapped tyrosinase (Figure S1), the ameliorative effect of liposome-entrapped tyrosinase against dopaminergic dysfunction could be due to the recovery of reduced DA levels following tyrosinase replacement. In the present study, furthermore, intrastriatal transplantation of melanogenic B16-F1 cells or primary cultured melanocytes from black mice showed marked improvement in abnormal contralateral rotation behavior in parkinsonian animals lasting up to 1–3 months after the transplantation, with significant increase in DA signals around the surviving transplant (Figures 2, 4 and 5). Freed et al. reported that B16-C3 melanoma cells transplanted into the striatum of normal rats expressed TH-immunoreactivity and catecholamine fluorescence by 3 weeks after transplantation [21]. Although intrastriatal transplantation of melanogenic retinal pigment epithelial cells failed to provide clinical benefits in parkinsonian patients [22], the amount of produced L-DOPA from the transplant is obscure in the clinical trial. In the present study, intrastriatal transplantation of non-melanogenic human hepatoma HLE cells transfected with tyrosinase cDNA (HLE/tyrosinase) into hemi-parkinsonian mice also markedly improved the asymmetric rotation behavior at day 14 up to 2 months after transplantation. The transplanted cells constitutively produced L-DOPA to increase the levels of L-DOPA and DA in the lesioned side of the striatum (Figure 3). On the other hand, transplantation of empty vector-transfected non-melanogenic HLE cells showed no significant ameliorating effects. Therefore, the therapeutic effects of transplantation of melanogenic cells into the striatum of hemi-parkinsonian animals could be explained by their tyrosinase-expressing properties to replace L-DOPA/DA levels.

Several neurotrophic factors, such as nerve growth factor, brain-derived neurotrophic factor and glial cell line-derived neurotrophic factor (GDNF), and/or those neurotrophic factors-secreting cell implants enhance function and survival of transplanted cells as well as remaining DA neurons in PD models [23], [24], [25], [26], [27]. Among these trophic factors, striatal expression of GDNF was examined, because it exerts neurotrophic and neuroprotective effects on DA neurons. No changes in GDNF expression were detected in the extracellular spaces around the melanocyte-transplants in the striatum of PD model mice (data not shown). However, it is hard to negate a possibility that the therapeutic effects of transplanted melanogenic cells in the present study may be, in part, due to possible trophic factor-secreting property of implanted cells.

Therapeutic efficacy of cell transplantation approaches for parkinsonian models or PD patients has been reported in the studies using various kinds of stem cells, neural precursor cells or other cells. However, those cell transplantation therapies have several possible adverse effects such as off-state dyskinesia, immune rejection and immunosuppressant use in allogeneic implants and tumor formation in stem cell implants [25], [28]. Furthermore, transplantation of fetal cells or embryonic stem cells has ethical and social concerns. The effects of intrastriatal transplantation of melanocytes obtained from the back skin in the present study imply potentially therapeutic benefits of autograft melanocyte transplantation in PD patients, because the autograft cell transplantation can avoid or reduce immunological, ethical and social concerns.

The transplantation of fetal mesencephalic tissue develops graft-induced dyskinesia (GID) in a subpopulation of PD patients. The GID is one of major complications referred to as off-medication dyskinesia that appears independent of L-DOPA therapy. Even if the autograft transplantation of melanocytes can reduce some complications or concerns of cell transplantation therapies, we will be unable to exclude a possible occurrence of GID in PD patients. Although mechanisms of GID are still obscure, postsynaptic supersensitivity prior to transplantation and DA receptor-mediated signaling may be involved in the development of GID as well as L-DOPA-induced dyskinesia [28], [29]. Furthermore, contaminated serotonergic neurons in the fetal tissue graft are thought to cause GID because GID is attenuated by serotonin 5-HT1A agonist [30]. However, the transplanted melanogenic cells were not contaminated with serotonergic neurons, and no apparent involuntary movements in PD models during at least observation periods in the present study. Therefore, melanocyte transplantation may ameliorate postsynaptic supersensitivity prone to develop GID.

Since a toxic molecule of DA quinone could be generated in the oxidation of excess DA by tyrosinase, it might be argued that DA from transplanted tyrosinase-containing melanocytes would be converted to DA quinones that exert neurotoxicity. However, our previous studies revealed that tyrosinase could enzymatically and rapidly oxidize excess amounts of cytosolic DA to form stable melanin, which may rather prevent the slow progression of cell damage induced by DA auto-oxidation [17], [31]. If DA level in the striatum was excess, furthermore, the transplanted animals would show ipsilateral rotation behavior after apomorphine injection. In fact, sham-operated normal animals transplanted with genetically tyrosinase-overexpressed HLE cells showed rotation behavior towards the rather ipsilateral side (Figure 3). However, all intrastriatal transplantation of tyrosinase-containing cells to hemi-parkinsonian animals ameliorated the apomorphine-induced rotation behavior towards the contralateral side, but caused no involuntary ipsilateral rotation in the present study (Figures 2, 3, 4 and S1). Therefore, the transplantation of tyrosinase-containing melanocytes in this experiment supplemented DA, but it would not render DA levels excess in the lesioned-side of the striatum up to 1–3 months after transplantation. In other words, DA levels may be well controlled for at least 3 months after the melanocyte transplantation. Further long-term examination would be needed to evaluate possible auto-regulating effect of implanted melanocytes on DA levels in parkinsonian models.

In conclusion, we revealed that intrastriatal transplantation of tyrosinase-expressing melanocytes from the back skin brought long-lasting amelioration of the apomorphine-induced abnormal rotation behavior in hemi-PD models. The present results highlight potentially therapeutic benefits of intrastriatal autograft cell transplantation of tyrosinase-expressing melanocytes in PD patients, because the autograft cell transplantation can avoid or reduce immunological, ethical and social concerns.

## Supporting Information

Figure S1 **Effects of tyrosinase intrastriatal lipofection in hemi-parkinsonian rats.** (A) Rotation behavior towards contralateral side for 2 min at 10 min after apomorphine injection (0.1 mg/kg, s.c.) was recorded at 3, 6 and 12 days after lipofection of liposome-entrapped tyrosinase (19.2 U/4 µg/4 µl lipofectin X2) or lipofectin alone into the right lateral striatum of hemi-parkinsonian rats or sham-operated controls. Values are means ± SEM of 3–4 rats. *p<0.05, ***p<0.001 vs. each sham-lipofectin, ^++^ *p*<0.01, ^+++^ *p*<0.001 vs. each 6-OHDA-lipofectin, ^###^ *p*<0.001 vs. each preinjection of tyrosinase. Representative photographs of striatal tyrosinase-IR (B) and DA-IR (C) in the tyrosinase-injected striatal area at day 14 after tyrosinase lipofection in hemi-parkinsonian rats. (B, C) Each right panel shows means integrated density of tyrosinase-IR or DA-IR ± SEM, respectively (n = 3–4). **p<0.01 vs. intact control side of each treated group. ^##^ *p*<0.0001 vs. side-matched lipofectin-injected group. ^+^ *p*<0.05 vs. side- and treatment-matched sham-operated group.
(TIF)

Figure S2 **Immunostaining of TH in the striatum (A) and the substantia nigra (B) in hemi-parkinsonian rats at day 14 after tyrosinase intrastriatal lipofection.** Quantitative data of integrated density of TH-IR in the right lateral striatal area (C) and substantia nigra pars compacta (D). Values are means ± SEM (n = 3). *p<0.05, **p<0.001 vs. intact control side of each treated group. ^++^ *p*<0.005 vs. side- and treatment-matched sham-operated group.
(TIF)

Figure S3 **TH-IR in the right lateral striatal area (A) and the substantia nigra (B) in hemi-parkinsonian rats at 52 days after intrastriatal transplantation of B16-F1 melanoma cells.** Quantitative data of integrated density of TH-IR in the striatum around the transplant (C) and substantia nigra pars compacta (D). Values are means ± SEM (n = 3–4). **p<0.0001 vs. intact control side of each treated group. ^++^ *p*<0.0001 vs. side- and treatment-matched sham-operated group.
(TIF)

Figure S4 **TH-IR in the right lateral striatum (A) and the substantia nigra (B) in hemi-parkinsonian albino mice at 88 days after intrastriatal transplantation of melanocytes obtained from albino (ICR) or black (C57BL) mice.** Quantitative data of integrated density of TH-IR in the right lateral striatal area around the transplant (C) and substantia nigra pars compacta (D). Values are means ± SEM (n = 3–4). **p<0.0005 vs. intact control side of each treated group. ^++^ *p*<0.0005 vs. side- and treatment-matched sham-operated group.
(TIF)

Movie S1 **Effects of intrastriatal injection of tyrosinase on apomorphine-induced rotation behavior in hemi-parkinsonian rats.** Representative movie of behavior at 10 min after apomorphine injection (0.1 mg/kg, s.c.) at 4 days before (pre-lipofection) and at 3 days after lipofection of liposome-entrapped tyrosinase (19.2 U/4 µg/4 µl lipofectin X2) or lipofectin alone into the right lateral striatum of hemi-parkinsonian rats or sham-operated controls.
(WMV)

Movie S2 **Effects of intrastriatal transplantation of B16-F1 melanoma cells on apomorphine-induced rotation behavior in hemi-parkinsonian rats.** Representative movie of behavior at 5 min after apomorphine injection (0.1 mg/kg, s.c.) on Day 16 after intrastriatal transplantation of B16-F1 melanoma cell (5×10^5^ cells/4 µl PBS X2) into the right lateral striatum of hemi-parkinsonian rats or sham-operated controls.
(WMV)

Movie S3 **Effects of intrastriatal transplantation of tyrosinase cDNA-transfected human hepatocytes HLE cells on apomorphine-induced rotation behavior in hemi-parkinsonian mice.** Representative movie of behavior at 5 min after apomorphine injection (0.5 mg/kg, s.c.) on Day 21 after the transplantation of HLE cells or HLE cells transfected with tyrosinase cDNA (1×10^6^ cells/2 µl PBS) into lesioned side of lateral striatum in hemi-parkinsonian albino mice or sham-operated controls.
(WMV)

Movie S4 **Effects of intrastriatal transplantation of primary cultured melanocytes on apomorphine-induced rotation behavior in hemi-parkinsonian mice.** Representative movie of behavior at 5 min after apomorphine injection (0.5 mg/kg, s.c.) on Day 14 after the transplantation of melanocytes from albino ICR or black C57BL mice (3×10^5^ cells/2 µl) or MDM medium alone into the right lateral striatum of hemi-parkinsonian albino mice.
(WMV)
