# Supplementary figures and images for: Transplantation of Melanocytes Obtained from the Skin Ameliorates Apomorphine-Induced Abnormal Behavior in Rodent Hemi-Parkinsonian Models
Source: PLoS One. 2013 Jun 12;8(6):e65983. doi: 10.1371/journal.pone.0065983 (PMC3680415; doi:10.1371/journal.pone.0065983)

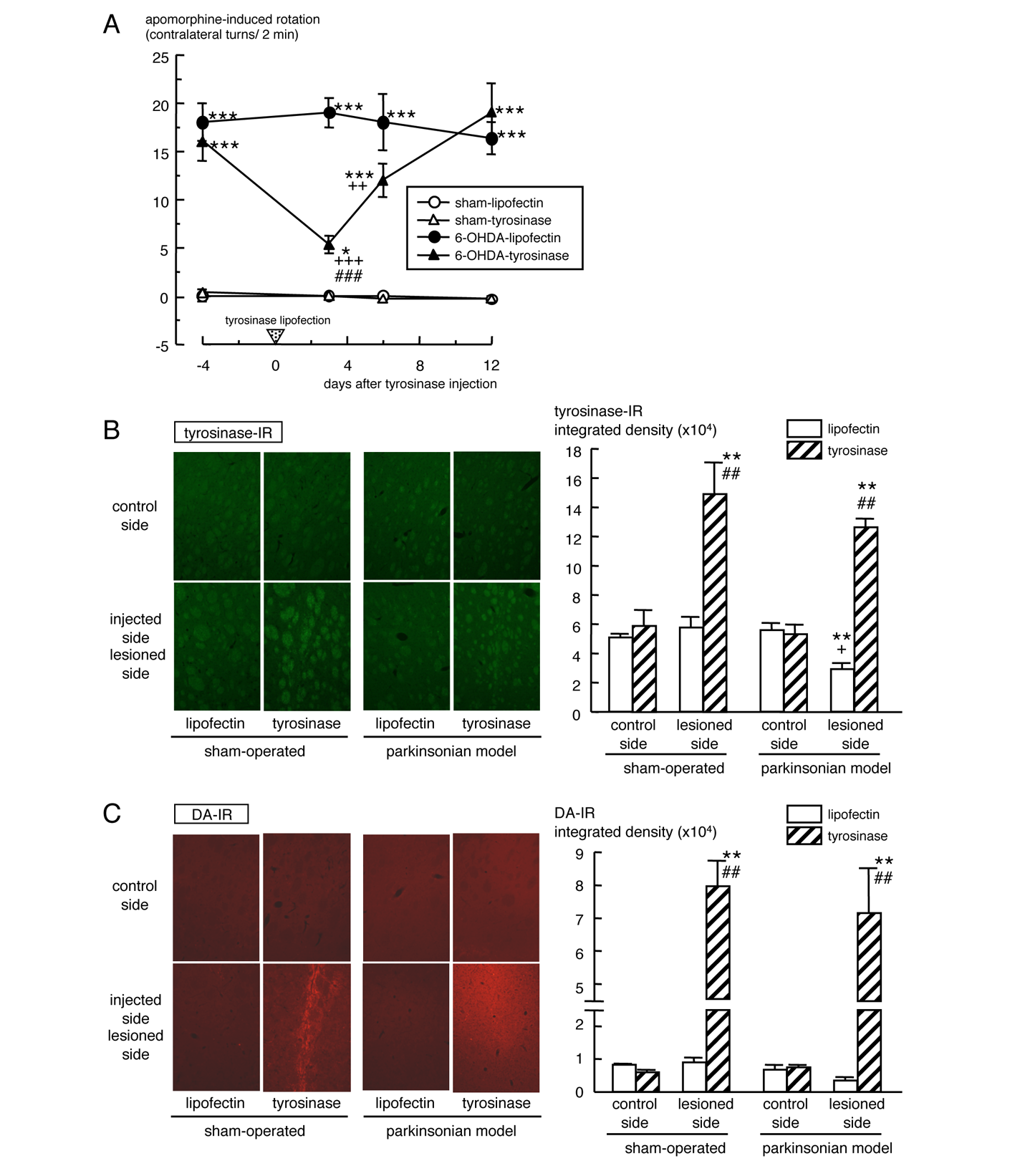

Supplement: Figure S1 — Effects of tyrosinase intrastriatal lipofection in hemi-parkinsonian rats. (A) Rotation behavior towards contralateral side for 2 min at 10 min after apomorphine injection (0.1 mg/kg, s.c.) was recorded at 3, 6 and 12 days after lipofection of liposome-entrapped tyrosinase (19.2 U/4 µg/4 µl lipofectin X2) or lipofectin alone into the right lateral striatum of hemi-parkinsonian rats or sham-operated controls. Values are means ± SEM of 3–4 rats. *p<0.05, ***p<0.001 vs. each sham-lipofectin, ++ p<0.01, +++ p<0.001 vs. each 6-OHDA-lipofectin, ### p<0.001 vs. each preinjection of tyrosinase. Representative photographs of striatal tyrosinase-IR (B) and DA-IR (C) in the tyrosinase-injected striatal area at day 14 after tyrosinase lipofection in hemi-parkinsonian rats. (B, C) Each right panel shows means integrated density of tyrosinase-IR or DA-IR ± SEM, respectively (n = 3–4). **p<0.01 vs. intact control side of each treated group. ## p<0.0001 vs. side-matched lipofectin-injected group. + p<0.05 vs. side- and treatment-matched sham-operated group. (TIF) [file pone.0065983.s001.tif]

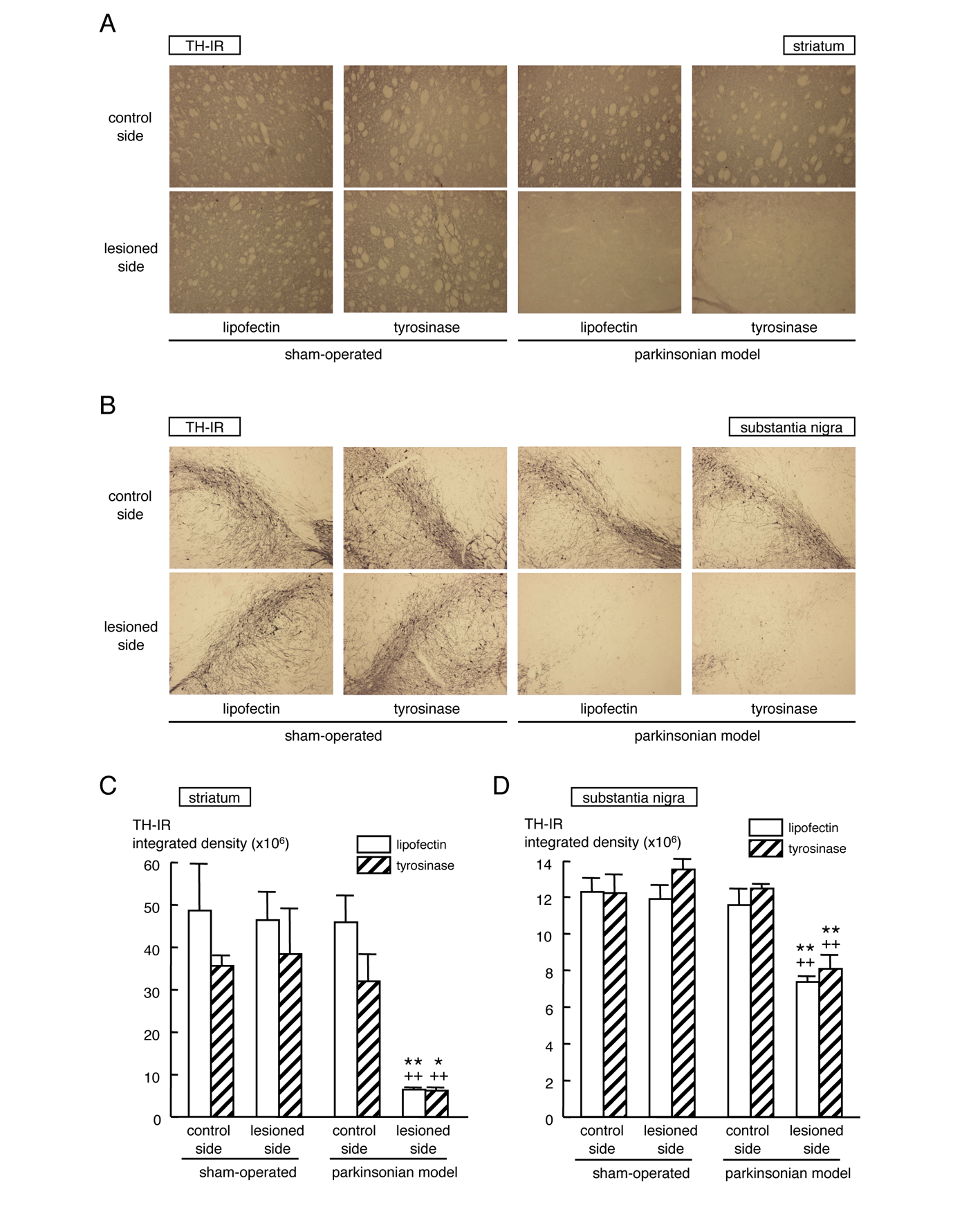

Supplement: Figure S2 — Immunostaining of TH in the striatum (A) and the substantia nigra (B) in hemi-parkinsonian rats at day 14 after tyrosinase intrastriatal lipofection. Quantitative data of integrated density of TH-IR in the right lateral striatal area (C) and substantia nigra pars compacta (D). Values are means ± SEM (n = 3). *p<0.05, **p<0.001 vs. intact control side of each treated group. ++ p<0.005 vs. side- and treatment-matched sham-operated group. (TIF) [file pone.0065983.s002.tif]

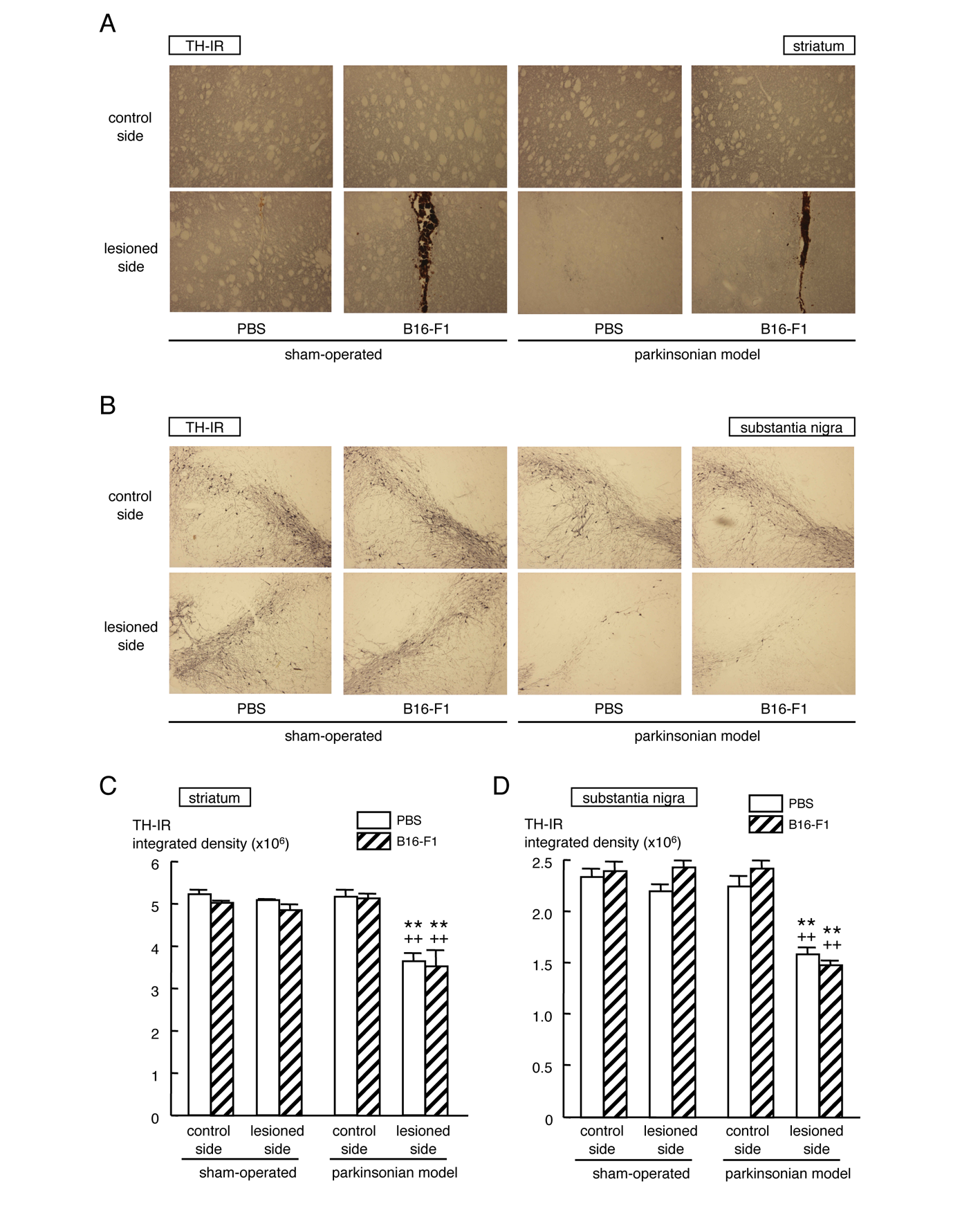

Supplement: Figure S3 — TH-IR in the right lateral striatal area (A) and the substantia nigra (B) in hemi-parkinsonian rats at 52 days after intrastriatal transplantation of B16-F1 melanoma cells. Quantitative data of integrated density of TH-IR in the striatum around the transplant (C) and substantia nigra pars compacta (D). Values are means ± SEM (n = 3–4). **p<0.0001 vs. intact control side of each treated group. ++ p<0.0001 vs. side- and treatment-matched sham-operated group. (TIF) [file pone.0065983.s003.tif]

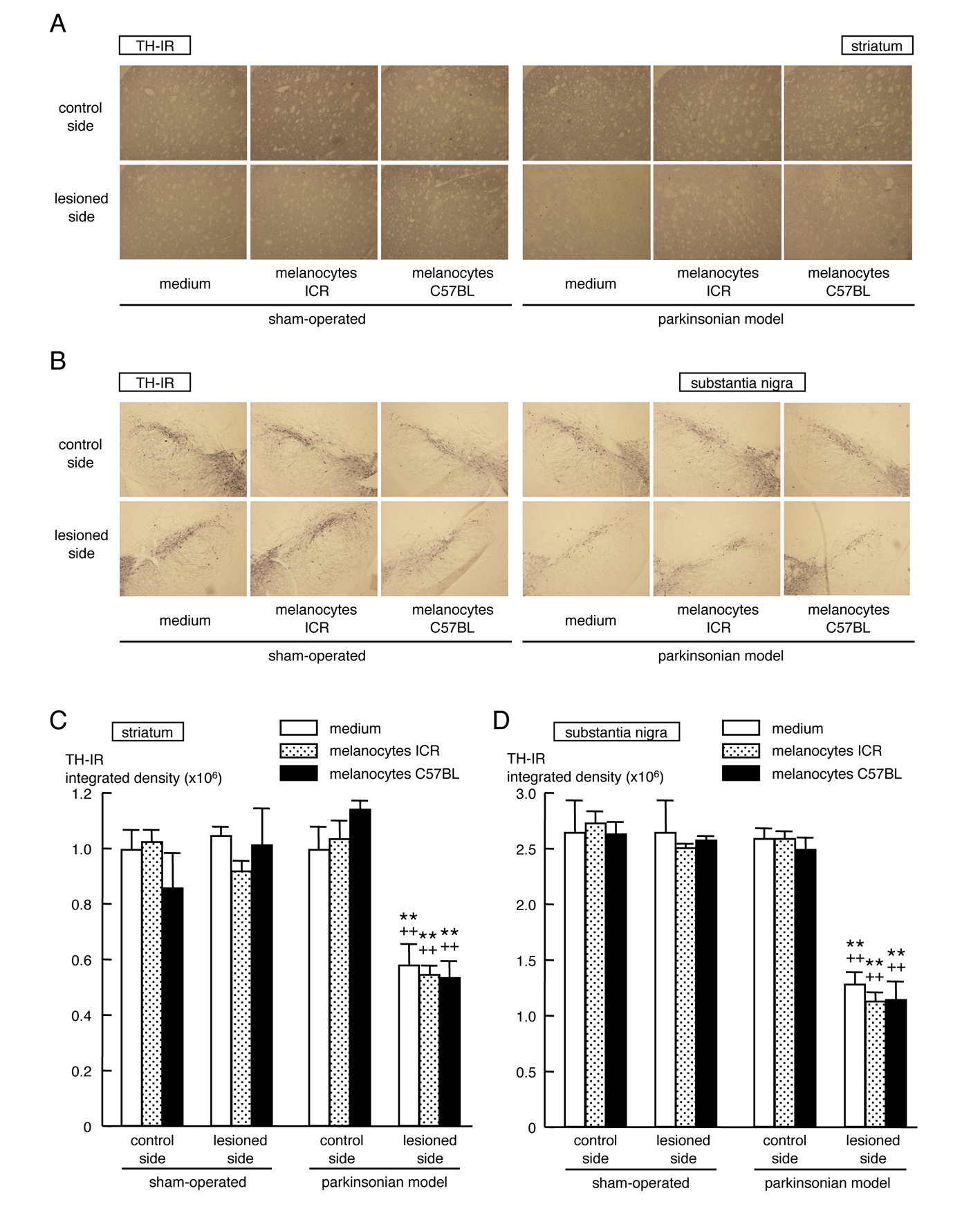

Supplement: Figure S4 — TH-IR in the right lateral striatum (A) and the substantia nigra (B) in hemi-parkinsonian albino mice at 88 days after intrastriatal transplantation of melanocytes obtained from albino (ICR) or black (C57BL) mice. Quantitative data of integrated density of TH-IR in the right lateral striatal area around the transplant (C) and substantia nigra pars compacta (D). Values are means ± SEM (n = 3–4). **p<0.0005 vs. intact control side of each treated group. ++ p<0.0005 vs. side- and treatment-matched sham-operated group. (TIF) [file pone.0065983.s004.tif]
